# Sleep Health among Community-Recruited Opioid-Using People Who Inject Drugs in Los Angeles, CA and Denver, CO

**DOI:** 10.1007/s11524-024-00922-3

**Published:** 2024-09-19

**Authors:** Avaion Ruth, Siddhi S. Ganesh, Pooja Shah, Erin E. Gould, Katrina Ninh, Rachel Carmen Ceasar, Dustin T. Duncan, Ricky N. Bluthenthal

**Affiliations:** 1 Department of Population and Public Health Sciences, Keck School of Medicine, University of Southern California, Los Angeles, CA USA; 2 Mailman School of Public Health, Department of Epidemiology, Columbia University, New York, NY USA

**Keywords:** Sleep health, Sleep duration, Sleep quality, People who use drugs, Structural vulnerability

## Abstract

Chronic insufficient and poor-quality sleep are linked to hypertension, diabetes, depression, heart attack, and stroke. While studies on substance use and sleep typically focus on people in or entering treatment, there is a lack of research on sleep health among community-recruited people who inject drugs (PWID). To address this literature gap, we examined factors associated with insufficient and poor-quality sleep among community-recruited PWID. We recruited and interviewed 472 active opioid-using PWID (injected within the last 30 days) in Los Angeles, CA and Denver, CO between 2021 and 2022. Participants completed computer-assisted interviews covering demographics, subsistence measures, drug use patterns, injection-related behaviors, health risks, and sleep duration and quality in the last 3 months. Descriptive statistics were used to analyze all variables for subjects with complete responses to sleep items (*n* = 464). Bivariate analyses determined factors associated with sleep measures using chi-square and *t*-tests. Collinear variables were removed, and binomial linear multivariable regression calculated risk ratios (RR) for insufficient and poor-quality sleep in the last 3 months. Participants exhibited low sleep duration (mean = 4.99, standard deviation (SD) = 2.70), with 76% reporting insufficient sleep and 62% poor-quality sleep. Bivariate analyses associated both sleep measures with drug use, high subsistence scores, violent victimization, and poor health outcomes. Multivariable analyses showed a high subsistence score predicting insufficient (RR = 1.31) and poor-quality sleep (RR = 1.69) compared to low subsistence. Poor sleep health is common among structurally vulnerable community-recruited PWID, as measured by subsistence index associated with adverse sleep outcomes. Further research on structural interventions to address sleep and subsequent health outcomes among PWID is imperative.

## Introduction

People who inject drugs (PWID) are a vulnerable population that face unique health challenges. Insufficient sleep can weaken the immune system, potentially increasing vulnerability to infections, including viral hepatitis, conditions that disproportionately impact PWID [1].

There is also an emerging body of evidence associating substance use with sleep health issues [1–4]. A study that compared people with substance use disorders (SUDs) to those without reported that poor sleep was more prevalent among people with SUD, specifically those with opioid use disorder and cannabis use disorder [4]. Among vulnerable populations, a study that sampled men who have sex with men reported that poor sleep quality was associated with alcohol and marijuana use, while short sleep duration was associated with methamphetamine use [3]. Both short duration and poor quality of sleep were associated with depressive symptoms [3].

Dubar [5] referred to sleep as a “biosocial marker of social justice” wherein sleep health can be an indicator that reflects both biological and social dimensions of inequality [5]. Examining sleep through such a framework allows it to serve as an observable and measurable domain that informs us of the intersection of biological factors (as a determinant of health) and social factors (as access to sleep is socially determined). Social-structural conditions enable and incentivize the displacement of people who use drugs. People who use drugs face disproportionate rates of housing and financial insecurity [6]. In a study sampling recently evicted people who use drugs, participants identified a range of financial and safety-related concerns tied to poor sleep [6]. Primary drivers of poor sleep included fear of theft, city worker and police sweeps, sexual and gender-based violence, and oppressive shelter conditions [6]. A recent study sampling a suburban sample of people who use opioid identified structural vulnerabilities, such as hunger and homelessness, as statistically significant correlates of sleep impairments measured via sleep contexts, problems, and schedules [7].

Despite the well-documented effects of chronic sleep disturbances on health, our understanding of the specific impact of sleep deprivation on certain populations, particularly community-recruited PWID, remains limited. Previous research in this area has predominantly focused on individuals in treatment settings leaving significant gaps in our knowledge regarding the sleep health of PWID [8]. Additionally, studies that have examined structural vulnerabilities have examined smaller sample sizes [7]. To address these research gaps, we explore subsistence and substance use factors among others to inadequate sleep and poor sleep quality, aiming to understand factors impacting sleep health among opioid-using PWID in Los Angeles, CA and Denver, CO.

## Methods

### Sampling and Recruitment

We recruited PWID from community settings (syringe services programs, homeless service facilities, and areas with open drug scenes) between April 2021 and November 2022 in Denver, Colorado and Los Angeles, CA. Eligibility criteria for the study were self-reported age of 18 or older, visual evidence of recent drug injection, and self-reported recent drug injection and opioid use (including heroin, fentanyl, and/or prescription opioids). Participants completed a survey that included the following domains: demographic and socioeconomic characteristics, drug use patterns including types, routes of administration and frequency, health conditions, exposure to violence and police/security guards, and sleep health among other items. Survey data were collected during a one-on-one session with a trained interviewer, and data were recorded using computer-assisted personal interview software (Questionnaire Development System, Nova Research, Bethesda, MD). Participants received $20 for completing this interview. This cross-sectional analysis includes data from 472 PWID, of which 223 from Los Angeles and 249 from Denver. All study procedures were reviewed and approved by the institutional review board at the University of Southern California.

### Study Measures

Sleep health was assessed using the 2 items from the Pittsburgh Sleep Quality Index (PSQI) to minimize participant burden as sleep measures were not a primary aim of the study. Participants were asked, “In the last 3 months, how would you rate the quality of your sleep?” with response options ranging from “very bad” to “very good” on a 4-point Likert scale. Those reporting “fairly bad” or “very bad” sleep quality in the last 3 months were classified as having poor-quality sleep. Participants were then asked: “In the last 3 months, how many hours of actual sleep do you usually get per day?” Those reporting less than 7 h of sleep on average were classified as having insufficient sleep per CDC guidelines (Centers for Disease Control and Prevention [CDC]).

We considered covariates from the following domains: socio-demographic and socioeconomic characteristics, drug use patterns, health measures including conditions and use of preventative and medical services, and contact with violence and police. Among demographic and socioeconomic items, we considered race (White, Black, Latinx, Asian/Pacific Islander, Native American, and Mixed), gender (male, female, or transgendered), and age (under 40 years old and 40 years old and above). Socioeconomic measures included housing instability (housed, unhoused and not displaced, and unhoused and displaced), educational attainment (high school or more), monthly income (< $1000, $1001 to $1400, 1401 to $2100, or $2101 or more), income sources in the last 3 months (job, unemployment, veteran’s benefits, welfare, disability, social security, spouse, family, friends, recycling, panhandling, and illegal or possibly illegal sources), and subsistence items and index [9]. The subsistence items consisted of 5 questions that address the difficulty (from never to usually) of obtaining essential things (food, shelter, clothing, access to shower, and toilets). These items were considered individually in this analysis and as an index where the sum score for each participant was calculated and then divided into terciles leaving a categorical measure from low to high where a high score indicates greater difficulty obtaining these essential things.

We assessed drug use in the last 3 months for the following drugs: cocaine, crack cocaine, methamphetamine, heroin, speedball (admixture of cocaine and heroin), goofball (admixture of heroin and methamphetamine), and non-medical use of prescription opiates, stimulants, sedatives, tranquilizers, methadone, and buprenorphine. For each drug, participants reported use in the last 3 months and how often they used it through injection and non-injection. From these responses, we examined use as (1) any in the last 3 months and (2) use frequency (none, less than daily, once or twice a day, or 3 or more times a day).

We considered health measures including diagnosis with HIV, HCV, diabetes, high blood pressure, and other common chronic health conditions among PWID. In Table 1, we reported health variables that had a statistically significant difference for at least one of the sleep measures. HIV, HCV, high blood pressure, and other health conditions were not significantly different for either insufficient sleep or poor-quality sleep. In addition, participants were asked about whether they had any withdrawal symptoms related to opioid, methamphetamine, and cocaine use in the last 3 months using a previously published measure [10]. We also collected information on how participants regarding their drug use and involvement in substance use treatment using the following items: (1) “My drug use is a problem for me,” (2) “My drug use is more trouble than it is worth,” and (3) “My drug use is under control.” Response options were “strongly disagree” to “strongly agree” on a five-point Likert scale. For drug treatment, participants we asked about current and enrollment in the last 3 months in the following types of substance use treatment: methadone maintenance, methadone detoxification, buprenorphine maintenance, buprenorphine detoxification, vivitrol, outpatient, inpatient/hospitalization, and residential treatment.

**Table 1:** Selected demographic, socioeconomic, drug use, and health characteristics of opioid-using people who inject drugs by sleep health variables in Denver, CO and Los Angeles, CA, 2021/22 (*n* = 464). *p < 0.05

Characteristics	Total (*N* = 464) *N* (%)	Insufficient sleep (*N* = 354) *N* (%)	Poor quality sleep (*N* = 289) *N* (%)
Demographics			
City			
Denver	246 (53%)	196 (55%)	152 (53%)
Los Angeles	218 (47%)	158 (45%)	137 (47%)
Gender			
Male	358 (77%)	280 (79%)	224 (78%)7
Female	98 (21%)	69 (19%)	60 (21%)
Transgender/non-binary/other	8 (2%)	5 (2%)	5 (2%)
Race			
White	242 (52%)	184 (52%)	145 (50%)
Latinx	121 (26%)	97 (27%)	85 (29%)
African American	26 (6%)	20 (6%)	15 (5%)
Asian/Pacific Islander	5 (1%)	3 (1%)	4 (2%)
Native American	47 (10%)	33 (9%)	26 (9%)
Other	23 (5%	17 (5%)	14 (5%)
Age			
< 40	236 (51%)	179 (51%)	160 (55%)
40 or more	228 (49%)	175 (49%)	129 (45%)*
Heterosexual	374 (81%)	286 (81%)	239 (83%)
High school education or more	358 (77%)	269 (76%)	216 (75%)
Socioeconomics			
Housing/displacement in the last 3 months			
Housed	97 (21%)	65 (18%)	50 (17%)
Unhoused, no displacement	74 (16%)	54 (15%)	43 (15%)
Unhoused and displaced	293 (63%)	235 (66%)*	196 (68%)*
Places stayed, last 3 months			
Tent	282 (61%)	223 (63%)	184 (64%)
Outdoors (not tent)	297 (64%)	240 (68%)*	202 (70%)*
Vehicle	159 (34%)	126 (36%)	117 (41%)*
Abandoned building/garage/shed	156 (34%)	128 (36%)*	111 (38%)*
Own house/apt/hotel room	180 (39%)	130 (37%)	111 (38%)
Temporary hotel	139 (30%)	113 (32%)	95 (33%)
Rented room	76 (16%)	58 (16%)	51 (18%)
With family, friend, sexual partner	164 (35%)	131 (37%)	106 (37%)
Shelter	69 (15%)	52 (15%)	43 (15%)
Hospital	112 (24%)	91 (26%)	74 (26%)
Jail/prison	67 (14%)	53 (15%)	47 (16%)
Subsistence measures			
Difficulty finding shelter			
Never	203 (44%)	133 (38%)	100 (35%)
Rarely	47 (10%)	39 (11%)	26 (9%)
Sometimes	88 (19%)	72 (20%)	66 (23%)
Usually	126 (27%)	110 (31%)*	289 (34%)*
Difficulty getting enough to eat			
Never	203 (44%)	141 (40%)	99 (34%)
Rarely	56 (12%)	45 (13%)	40 (14%)
Sometimes	117 (25%)	91 (26%)	80 (28%)
Usually	88 (19%)	77 (22%)*	70 (24%)*
Difficulty finding clothing			
Never	221 (48%)	153 (43%)	116 (40%)
Rarely	38 (8%)	31 (9%)	22 (8%)
Sometimes	97 (21%)	77 (22%)	69 (24%)
Usually	108 (23%)	93 (26%)*	82 (28%)*
Difficulty finding place to wash up			
Never	177 (38%)	126 (36%)	93 (32%)
Rarely	36 (8%)	29 (8%)	19 (7%)
Sometimes	97 (21%)	70 (20%)	65 (22%)
Usually	154 (33%)	129 (36%)*	112 (39%)*
Difficulty finding restroom			
Never	166 (36%)	108 (31%)	79 (27%)
Rarely	28 (6%)	21 (6%)	15 (5%)
Sometimes	102 (22%)	82 (23%)	71 (25%)
Usually	168 (36%)	143 (40%)*	124 (43%)*
Subsistence index			
Low	144 (31%)	95 (27%)	67 (23%)
Medium	163 (35%)	123 (35%)	98 (34%)
High	155 (34%)	134 (38%)*	122 (43%)*
Monthly income			
< $1000	245 (53%)	188 (53%)	148 (51%)
$1001 to $1400	87 (19%)	66 (19%)	56 (19%)
$1401 to $2100	61 (13%)	48 (14%)	41 (14%)
$2101 or more	67 (15%)	49 (14%)	43 (15%)
Income source, last 3 months			
Job	100 (22%)	75 (21%)	62 (22%)
Unemployment	48 (10%)	34 (10%)	27 (9%)
VA benefits	5 (1%)	4 (1%)	3 (1%)
Welfare, general relief	223 (48%)	173 (49%)	143 (50%)
SSDI/state disability	32 (7%)	21 (6%)	15 (5%)
SSI/retirement benefits	38 (8%)	22 (6%)*	15 (5%)*
Family/spouse	93 (20%)	71 (20%)	62 (22%)
Recycling	109 (24%)	85 (24%)	65 (23%)
Illegal or possibly illegal source	246 (53%)	199 (56%)*	172 (60%)*
Panhandling	161 (35%)	132 (37%)*	109 (38%)
Drug use			
Cocaine use, last 3 months			
None	222 (48%)	163 (46%)	125 (43%)
Less than daily	207 (45%)	161 (46%)	135 (47%)
One to 2 times a day	18 (4%)	15 (4%)	14 (5%)
3 times a day or more	17 (4%)	15 (4%)	15 (5%)*
Drug use, last 3 months			
Crack cocaine	154 (33%)	123 (35%)	102 (35%)
Powder cocaine	143 (31%)	108 (31%)	94 (33%)
Methamphetamine	387 (83%)	306 (86%)*	247 (86%)
Speedball	135 (29%)	111 (31%)	97 (34%)*
Goofball	292 (63%)	233 (66%)*	186 (64%)
Fentanyl	326 (70%)	254 (72%)	206 (71%)
Heroin	380 (82%)	288 (81%)	240 (83%)
Non-prescription use			
Opioids	131 (28%)	102 (29%)	87 (30%)
Tranquilizers	174 (38%)	137 (39%)	122 (42%)*
Stimulants	59 (13%)	46 (13%)	42 (15%)
Methadone	73 (16%)	61 (17%)	56 (19%)*
Cannabis	349 (75%)	270 (76%)	215 (74%)
Methamphetamine use, last 3 months			
None	53 (11%)	31 (9%)	28 (10%)
Less than daily	140 (30%)	109 (31%)	87 (30%)
One to 2 times a day	155 (33%)	124 (35%)	101 (35%)
3 times a day or more	116 (25%)	90 (25%)*	73 (25%)
My drug use is a problem (*n* = 469)			
Strongly disagree	20 (4%)	16 (5%)	9 (3%)
Disagree	43 (9%)	29 (8%)	18 (6%)
Neutral	56 (12%)	39 (11%)	24 (8%)
Agree	173 (38%)	135 (38%)	118 (41%)
Strongly agree	169 (37%)	133 (38%)	119 (41%)*
My drug use is more trouble than it is worth			
Strongly disagree	24 (5%)	18 (5%)	11 (4%)
Disagree	80 (17%)	58 (17%)	38 (13%)
Neutral	52 (11%)	36 (10%)	25 (9%)
Agree	164 (36%)	133 (38%)	119 (42%)
Strongly agree	141 (31%)	107 (30%)	94 (33%)*
My drug use is under control			
Strongly disagree	82 (18%)	69 (20%)	62 (22%)
Disagree	168 (37%)	130 (37%)	117 (41%)
Neutral	76 (17%)	53 (15%)	38 (13%)
Agree	112 (24%)	82 (23%)	59 (21%)
Strongly agree	22 (5%)	18 (5%)	11 (4%)*
Health/violence/other			
Substance use treatment in the last 3 months			
Methadone maintenance	93 (20%)	79 (22%)*	62 (22%)
Ever diagnosed with			
Diabetes	30 (7%)	26 (7%)	24 (8%)*
Withdrawal symptoms, last 3 months			
Opioids	346 (75%)	266 (75%)	226 (78%)*
Methamphetamine	190 (41%)	150 (42%)*	120 (42%)
Cocaine	21 (5%)	19 (5%)	18 (6%)*
Criminal legal contact, last 3 months			
Police	201 (43%)	160 (45%)	135 (47%)
Security guard	220 (48%)	181 (51%)*	153 (53%)*
Violence, last 3 months			
Threatened with weapon	179 (39%)	148 (42%)*	129 (45%)*
Physically hurt	153 (33%)	136 (38%)*	117 (41%)*
Weapon used against you	122 (26%)	100 (28%)	93 (32%)*
Coerced sex	26 (6%)	24 (7%)*	20 (7%)
Belongings stolen	356 (77%)	284 (80%)*	233 (81%)*
Stranger attack on the streets	125 (27%)	110 (31%)*	96 (33%)*
Violence exposure count			
None	88 (19%)	60 (17%)	46 (16%)
One	151 (33%)	107 (30%)	82 (28%)
Two to three	110 (24%)	84 (24%)	71 (25%)
Four or more	115 (25%)	103 (29)*	90 (31%)*

Finally, we collected information on violence exposure and contact with police and security guards. Our violence items asked about any threats with a weapon, attacks by slapping or kicking, attacks with weapon, sexual coercion, having belongings stolen, and attacks by a stranger for the last 3 months (yes or no). We also summed these items to create a violence index based on tercile of sum score (low, medium, and high). Lastly, participants we asked about any contact with police, security guards, and criminal legal system (imprisonment, parole, or probation) in the last 3 months.

### Statistical Analysis

For each variable, descriptive statistics (e.g., frequencies, means, standard deviations, among others) were examined. We conducted bivariate analyses using Pearson’s chi-square for categorical variables and *t*-test for continuous variables to identify factors associated with insufficient sleep and poor-quality sleep, separately. Statistical significance of bivariate comparisons was set at *p* < 0.05. Collinear variables within domains (demographics, socioeconomic, drug use types, health, and violence and police) were removed from the final analysis based on strength of association with the dependent variable. Binomial linear regression was used to determine risk ratios (RR) for insufficient sleep and poor-quality sleep in the last 3 months separately. All quantitative data analysis was conducted with SPSS version 29.01.

## Results

The study consisted of 472 participants (249 from Denver and 223 from Los Angeles), of which 77% were male, 52% were White, 26% were Latinx, 10% were Native American, and 6% were Black. Participants were less than 40 years of age (51%) and 40 years of age and older (49%).

Sleep health measures were poor overall for the sample, with 76% reporting insufficient sleep (mean hours = 5; standard deviation = 2.69, and median = 5) and 62% reporting “very” or “fairly” bad sleep quality. Table 1 provides bivariate results by insufficient sleep and poor-quality sleep. Each measure of sleep was associated with socioeconomic variables (i.e., subsistence items, sleeping location, and income sources), drug use variables (especially methamphetamine and prescription tranquilizers and methadone), and opioid withdrawal symptoms.

We constructed two models predicting insufficient sleep and poor-quality sleep using binomial generalized regression (Table 2). Table 2 includes confidence intervals and *p*-values for the subsistence measure associated with both sleep measures. The other variables did not remain significant when included in the model with subsistence variable, and so, we only reported results for the subsistence index. We now refer to the outcome statistics as “risk ratios” since we did not adjust for other variables. For each model, high subsistence was associated with our outcomes (insufficient sleep, risk ratio (RR) = 1.31, 95% confidence interval (CI) = 1.01, 1.70; poor-quality sleep, RR = 1.69; 95% CI = 1.26, 2.28) as compared to low subsistence index.

**Table 2:** Separate binomial generalized regression model of insufficient sleep and poor quality sleep in the last 3 months among PWID in Denver, CO and Los Angeles, CA 2021/22

	Insufficient sleep Risk ratio (95% confidence interval)	*p* =	Poor quality sleep Risk ratio (95% confidence interval)	*p* =
Subsistence index				
Low	Referent		Referent	
Medium	1.14 (0.88, 1.50)	0.33	1.29 (0.95, 1.76)	0.10
High	1.31 (1.01, 1.70)	0.04	1.69 (1.26, 2.28)	< 0.001

## Discussion

In this study, we sought to identify factors associated with poor quality sleep and insufficient sleep among community-recruited and opioid-using PWID. Participants with poor quality sleep and insufficient sleep were statistically significantly more likely to report difficulty finding shelter, food, clothing, showers, and toilets. Consequently, among people with poor-quality sleep and in insufficient sleep, high subsistence scores (where a high score indicates greater difficulty accessing essential items and services) were observed at statistical significance (Table 1). Extant literature has established that food insecurity is associated with poor sleep quality, trouble falling and staying asleep, frequent insufficient sleep, shorter sleep duration, sleep disorders in adults, and access to healthcare [11, 12]. Given these findings, it is likely that PWID experiencing poor quality and insufficient sleep also face significant challenges in accessing essential items and services, mirroring the established link between food insecurity and sleep issues in other marginalized populations [11]. In both models, only high subsistence, or difficulty accessing essential items and services, was associated with our outcomes as compared to a low subsistence index, emphasizing the relationship between structural vulnerabilities and sleep health (Table 2). Other studies have found that struggles obtaining basic needs to influence health, particularly being unhoused. Housing instability among PWID has been associated with injection risk behaviors such as receptive syringe sharing, syringe mediated sharing, equipment sharing [13], and increased drug-related morbidity and mortality [14]. Furthermore, a recent study sampling people who inject drugs in Tijuana and San Diego found insecurity with water, sanitation, and hygiene (WaSH) to be associated with anxiety among PWID [15]. Our results suggest that both WaSH, housing and food insecurity, should be addressed in efforts to improve well-being and health among PWID.

Contrary to our expectations, drug use patterns were not associated with sleep health. Rather, the personal circumstances related to social determinants of health appear more salient for this outcome [16]. While drug use can influence an individual’s capacity to obtain basic needs, it is also influenced by structural vulnerabilities. For people who use drugs, debilitation of the capacity to obtain basic needs comes from varied sources including incarceration, convictions, rules restricting access to public goods (like housing, food assistance, and educational opportunities), and stigma [17]. Along with efforts to improve access to substance use treatment, interventions to improve access to basic needs should be pursued for improving sleep health.

Key evidence or practice-based approaches to reducing subsistence needs, and subsequently improving sleep health, among PWID include Housing First interventions, universal basic income, and WaSH interventions. Housing First interventions (placing unhoused people in housing prior to addressing substance use, mental health, or other chronic problems) have been found to improve health and well-being among people who use drugs and alcohol [18]. Universal basic income pilots (where monthly stipends ranging from $500 USD to $1000 USD are provided to low-income individuals and families) have been found to improve health and well-being for recipients [19]. Some of these pilots have included unhoused and substance using individuals and reported improvements in transition to housing and reductions in food insecurity [20]. Likewise, there is growing evidence from other countries that providing people with direct access to WaSH facilities can improve gastrointestinal and respiratory health outcomes, education attainment and economic outcomes such as water and healthcare expenditure [21].

## Limitations

This research is not without limitations. First, as the data collected was self-reported, findings might have been impacted by recall and social desirability bias. However, items on injection risk, drug use patterns and practices, and health items have been found to have acceptable psychometric properties [22]. Second, the cross-sectional study design does not allow for causal inference. Despite this, this is a useful methodology for establishing preliminary evidence. This research may not be generalizable to PWID beyond Los Angeles, CA and Denver, CO. This study was conducted using convenience sampling methods as data collection occurred during the COVID-19 pandemic. While our findings may be generalizable to other suburban areas, and a study in Baltimore City and Anne Arundel County, Maryland [7] found similar associations, we are unable to make that determination due to our sampling strategy. To improve generalizability, future research should conduct targeted sampling in multiple urban areas.

Despite these limitations, this research provides valuable preliminary data on sleep health among PWID and important insights for the direction of future research in this understudied topic area. Future research should expand the geographic scope and sample size of the population studied to enhance generalizability. Further, as sleep health is essential to health in general, exploring how poor sleep impacts other health conditions often experienced by PWID like abscesses and other infections, chronic health conditions, and recovery from acute injuries and wounds is warranted. Lastly, research on prevention strategies that address basic needs in this population should be pursued to improve sleep health and other common concerns among PWID such as food insecurity, poor nutrition, and access to personal hygiene facilities [15].
